# Positional and temporal differences in peak match running demands of elite football

**DOI:** 10.5114/biolsport.2023.116006

**Published:** 2022-05-10

**Authors:** Bradley Thoseby, Andrew D Govus, Anthea C Clarke, Kane J Middleton, Ben J Dascombe

**Affiliations:** 1 Sport and Exercise Science, School of Allied Health, Human Services and Sport, La Trobe University; 2 High-Performance Department, Melbourne City Football Club, Melbourne, Australia; 3 Applied Sport Science and Exercise Testing Lab, University of Newcastle, Australia

**Keywords:** Team sports, Football, Soccer, Peak match running intensities, Peak running intensities

## Abstract

Temporal changes in the total running demands of professional football competition have been well documented, with absolute running demands decreasing in the second half. However, it is unclear whether the peak match running demands demonstrate a similar decline. A total of 508 GPS files were collected from 44 players, across 68 matches of the Australian A-League. GPS files were split into the 1^st^ and 2^nd^ half, with the peak running demands of each half quantified across 10 moving average durations (1–10 min) for three measures of running performance (total distance, high-speed distance [> 19.8 km **·** h^−1^] and average acceleration). Players were categorised based on positional groups: attacking midfielder (AM), central defender (CD), defensive midfielder (DM), striker (STR), wide defender (WD) and winger (WNG). Linear mixed models and effect sizes were used to identify differences between positional groups and halves. Peak running demands were lower in the second half for STR across all three reported metrics (ES = 0.60–0.84), with peak average acceleration lower in the second half for DM, WD and WNG (ES = 0.60–0.70). Irrespective of match half, AM covered greater peak total distances than CD, STR, WD and WIN (ES = 0.60–2.08). Peak high-speed distances were greater across both halves for WIN than CD, DM and STR (ES = 0.78–1.61). Finally, STR had lower peak average acceleration than all positional groups across both halves (ES = 0.60–1.12). These results may help evaluate implemented strategies that attempt to mitigate reductions in second half running performance and inform position specific training practices.

## INTRODUCTION

Traditionally the physical demands of professional football match play have been quantified as absolute match running volumes using either video match analysis systems or global positioning systems (GPS) [1, 2]. As such, the physical demands of football and the intra- and inter-match differences in physical match demands (total distance (TD), high-speed distance [> 19.8 km · h^−1^] (HSD) and acceleration profiles) have become routinely reported [2–4]. Separately, research has explored the effects of acute reductions in physical performance during match-play, as represented by temporal changes in physical demands throughout a match [3]. Recent methods have quantified changes in physical outputs across discrete periods of match-play, such as between halves or distinct 15 min periods within halves e.g., 0–15 min, 15–30 min [5–7]. Collectively, this research has demonstrated that physical output is reduced during the second half, with the greatest reduction occurring in the final 15 minutes of the match [8]. Interestingly, this reduction during the final stages of a match coincides with a reported increase in goal scoring opportunities [9–12]. Consequently, the ability to perform at higher intensities for shorter periods when fatigued appears crucial to creating or denying goal scoring opportunities [11, 12]. As such, understanding the match demands and how these fluctuate throughout a match can help inform training practices to prepare players for competition.

Quantifying the physical demands of match-play provide context for training prescription and preparing players for competition. Recent methods have explored the most physically demanding periods of match-play through quantifying the peak match running demands for a range of short epochs rather than absolute running volumes for arbitrary epochs [13–15]. These peak match running demands are calculated using a moving average duration, where the highest observed physical output for selected metrics are calculated at incremental discrete time periods (e.g. 1–10 min), irrespective of match time [13]. Through calculating the peak match intensities, the most physically demanding periods of a match can be identified and replicated through training and conditioning drills. For example, the peak distance covered over 5 min during a match could be used to inform a training drill of 5 min in duration. Importantly, the use of a rolling average (e.g. 0–1, 0.1–1.1, 1.2–2.2 min) has been shown to be more accurate than assessing peak match running demands using predefined 1 min periods (e.g. 0–1, 1–2, 2–3 min), with the predefined periods underestimating peak match running demands by ˜7–25% [14]. Such variance between analysis techniques reflects that the peak match running demands often traverse multiple predefined periods, meaning that performance staff may interpret match demands and prescribe training below match intensities [16, 17].

With football being stochastic in nature and players frequently required to perform at are near maximal intensities, the mechanisms underlying acute changes to physical performance are multi-factorial and require a holistic approach to quantify [18, 19]. As such, monitoring physical performance data may not only be used to inform training practices, but also to help assess both individual and team physical performance and acute change to physical demands during match play [8]. One such important consideration is a players position, with position related differences observed for both total match outputs [20] and peak match running demands [13]. Additionally, the available research strongly demonstrates that physical output declines between halves in football, with the magnitude of reductions being impacted by multiple factors including fatigue, the use of pacing strategies or differing match situations (Bradley & Noakes, 2013; Di Salvo et al., 2009; Mohr et al., 2003). However, there is limited research on changes in peak match running demands between halves, with only two papers reporting such differences [21, 22]. When all positional groups were pooled, reductions in peak total distance across the analysed time periods of 1, 3, 5 and 10 min were observed, with HSD (> 19.8 km **·** h^−1^) and sprint distance (> 25 km **·** h^−1^) being maintained between halves [22]. Similarly, when accounting for positional groups, Casamichana, Castellano [21] reported moderate reductions in peak total distance covered during the second half for central defenders (CD) across 3, 5 and 10 min window durations, with wide defenders (WD) and wingers (WIN) also having moderate reductions for the 10 min duration. Further, minimal differences were observed between halves for high metabolic load distance, with average metabolic power found to be reduced for all positional groups in the second half [21].

While position-related differences have been observed for both total match physical outputs [20] and peak match running demands [13], further assessment of between half differences by positional group is warranted. Of the single study that reported on the between half positional differences in peak match running demands [21], they included limited windows (1, 3, 5 and 10 min) and reported on total distance, high metabolic load distance and average metabolic power. As such, despite pooled positional data available in the literature [22], the between-half positional differences for peak match running demands of HSD and average acceleration (AveAcc) are yet to be reported on. As such, this study aimed to report on the within- and between-halves and positional groups differences in peak match running demands across a range of durations (1–10 min) for commonly assessed metrics to help progress match performance analysis and better inform training prescription.

## MATERIALS AND METHODS

An observational design was used to compare the positional differences in peak match intensities across incremental moving average between 1–10 minutes, between halves in elite football players. Data were collected from 44 professional footballers playing in the same team competing within the Australian Hyundai A-League. Data were collected across three seasons of competitive A-League matches, consisting of 68 matches, for a total of 508 individual match observations (13 ± 10 matches per player, range 1–43). With majority of peak match running demands occurring prior to the 70^th^ minute [23], data were only included if players participated in more than 70 minutes of a match. These observations were representative of the entire playing group, with match files categorised according to position, as Attacking Midfielder (AM; *n* = 6, files = 68), Central Defender (CD; *n* = 10, files = 118), Defensive Midfielder (DM; *n* = 6, files = 91), Striker (STR; *n* = 4, files = 46), Wide Defender (WD; *n* = 9, files = 105) and Winger (WNG; *n* = 9, files = 80); where the team in question typically used a 4-2-1-3 formation. Prior to collection of data, ethical approval was attained from La Trobe University (HREC#: 18056).

### Activity Profile

Player activity profiles were recorded during the entirety of match-play using 18 Hz (10 Hz GNSS) portable GPS units (STATSports, Northern Ireland) that were placed between the scapulae in a custom-made harness under the playing jersey. All GPS devices were turned on 30 min prior to match commencement to allow for satellite acquisition. The GPS units employed are valid and reliable in measuring locomotor speeds of team sport athletes [24, 25]. Following each match, GPS files were downloaded using proprietary software (STATSports, Northern Ireland) with the raw GPS files (inclusive of added time) were exported into statistical software (R Studio, v1.2.5033) for further analysis. The raw exported speed trace was filtered using a 4^th^ order one-way Butterworth filter with a cut-off frequency of 1 Hz. Individual data points, in which running speeds exceeded 10 m **·** s^−1^ and acceleration/deceleration values that exceeded 6 m **·** s^−2^ were classified as technical errors and replaced with zero values.

From the GPS data, three commonly assessed measures of running intensity were chosen for assessment: total distance covered, high-speed distance covered (> 19.8 km **·** h^−1^) and average acceleration, with total and high-speed distance made relative to playing time (m **·** min^−1^). Average acceleration was calculated through the summation of all absolute acceleration and deceleration speeds which were then averaged over a defined time duration to provide an indication of the total acceleration requirements of match-play [26]. Although the consolidation of both accelerations and decelerations into one variable may conceal the underlying mechanisms responsible for the load, it has been suggested that assessing both variables together will better reflect the intensity of the activity [16]. To quantify peak match intensities, a moving average technique was applied to all three of the match output variables for ten incremental time epochs (i.e. 1–10 min), with the maximum value from each epoch recorded and then fitted using a power law curve [13, 27].

### Statistical Analysis

Statistical analyses were conducted using R Studio statistical programming software (v1.2.5033, R Core Development Team, Vienna) using the *nlme* [28] and *lme4* [29] packages to conduct linear and nonlinear mixed effects analysis. Non-linear mixed models were used to calculate exponent and slope values for the power law model, with differences in peak match running demands of TD, HSD and AveAcc profiles between positions and halves assessed using linear mixed models. In the linear mixed model, fixed effects were included for intensity period, position [six levels: AM, CD, DM, STR, WD and WIN] and match halves (two levels: first half and second half). A random intercept was included for player and an exponential correlation structure, with a nugget effect, to account for temporal correlation between intensity periods. Linear mixed models were also used to assess between position and half measures of absolute TD, HSD and AveAcc, with a fixed effect included for match half (two levels: first half and second half). Raw unit differences between positional groups and halves, at each intensity period or half, were converted to standardised mean differences (SMD) by dividing the mean, raw unit difference by the within-subject standard deviation (SD) attained from the random effects (i.e., the square root of the residual variance term). The magnitude of the SMD was quantified using the following qualitative descriptors: trivial (<0.2), small (0.2-0.6), moderate (0.6-1.2), large (1.2-2.0), very large (2.0-4.0) and extremely large (>4.0) [30]. A worthwhile difference was determined as a moderate effect size (> 0.6), with the imprecision of model regression parameter estimates are expressed using 95% confidence intervals (CIs).

## RESULTS

**FIG. 1:**
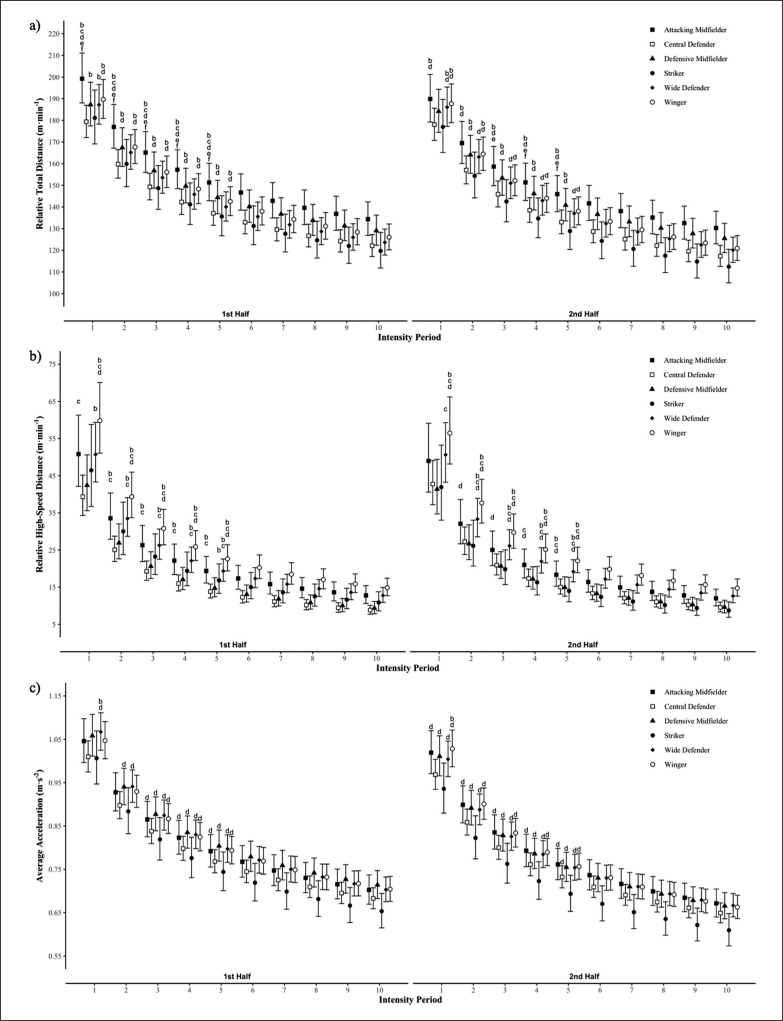
A positional comparison of peak match running demands of a) relative total distance, b) relative high-speed distance and c) average acceleration across each match half. Differences indicated if standardised mean difference is greater than 0.6. a = greater than Attacking Midfielder, b = greater than Central Defender, c = greater than Defensive Midfielder, d = greater than Striker, e = greater than Wide Defender, f = greater than Winger.

**FIG. 2:**
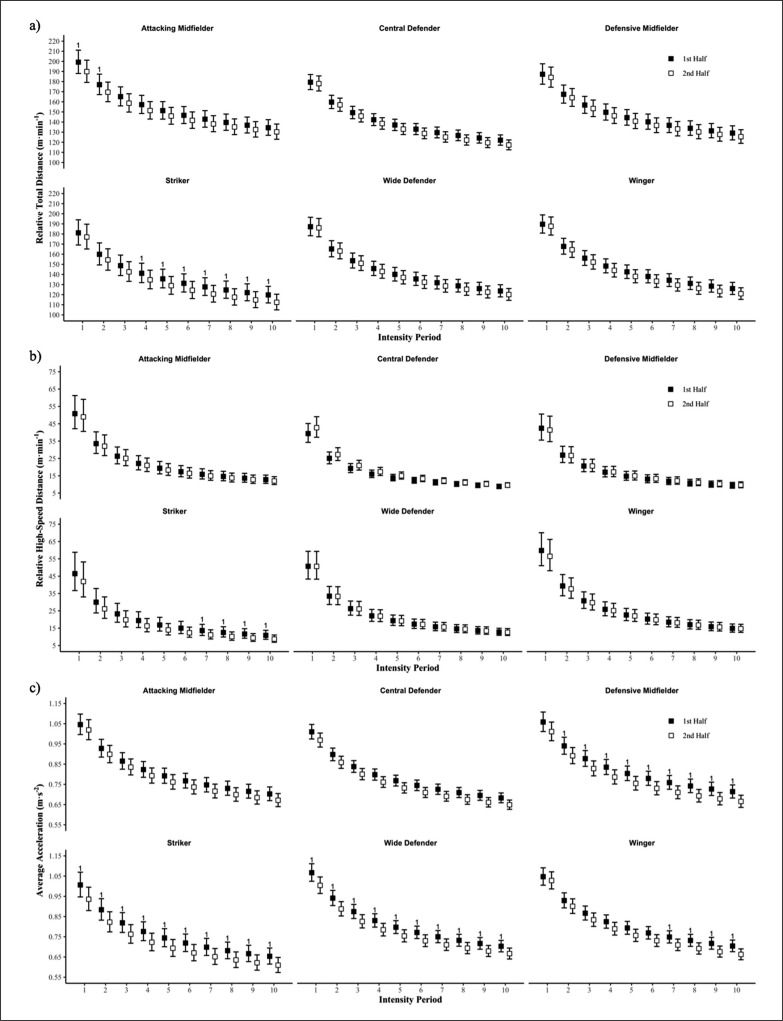
A between half comparison of peak match running demands of a) relative total distance, b) relative high-speed distance and c) average acceleration for each positional group. Differences indicated if standardised mean difference is greater than 0.6. 1 = greater than 2^nd^ half.

Absolute measures of total match physical output are presented in Table 1, with AM displaying higher absolute and relative TD than CD, WD and STR during the match (ES = 1.63–2.04). Further, WIN performed greater absolute HSD than all other positional groups, except AM (ES = 1.56–2.7). No positional differences were observed for AveAcc. The non-linear relationships of the peak match intensity power law models presented in Table 2, with differences between positional groups and halves presented in Figure 1. Attacking mid-fielders covered more relative TD than all other positional groups across the majority of epochs in the first half (ES = 0.62–1.63). Additionally, AM also had greater TD peak match running demands than all other positional groups except for DM for most epochs in the second half (ES = 0.70–2.08). Further, AM, WD and WIN had greater HSD peak match running demands than CD and DM across first half epochs (ES = 0.61–1.61), with CD, DM and STR all lower than AM, WD and WIN in the second half (ES = 0.60–1.61). Peak match intensities for AveAcc were similar in both halves amongst all positional groups, except for STR which were lower than all positional groups, except for CD in the first (ES = 0.63–1.02) and second (ES = 0.63–1.12) halves. Differences between halves for each positional group at each intensity period are presented in Figure 2. Minimal differences were observed between halves for relative TD and HSD, with the peak relative TD covered by STR reduced in the second half at the 4–10 min windows (ES = 0.60–0.89), and similarly for HSD for the 7–10 min windows (ES = 0.62–0.68). However, several differences were observed for AveAcc, with STR and WD having lower peak AveAcc demands for all peak intensity periods in the second half (ES = 0.62–0.84), with similar observations made for DM at the 2–10 min windows (ES = 0.61–0.81).

**TABLE 1:** Absolute and relative match outputs. Data presented as mean ± SD. Data in brackets indicate relative distances covered (m · min^-1^).

Variable		Attacking Midfielder	Central Defender	Defensive Midfielder	Striker	Wide Defender	Winger
**Match Duration (min)**	**1^st^ Half**	47 ± 1	47 ± 1	47 ± 1	47 ± 1	47 ± 1	47 ± 1
	**2^nd^ Half**	50 ± 1	50 ± 2	50 ± 2	49 ± 2	50 ± 2	50 ± 2
	**Match**	97 ± 2	97 ± 2	97 ± 2	96 ± 2	97 ± 2	97 ± 2

**Total Distance (m)**	**1^st^ Half**	5645 ± 362^b^^e^ (119 ± 10)^b^^e^	5070 ± 469 (107 ± 10)^l^	5261 ± 623 (111 ± 13)^l^	4742 ± 376 (101 ± 8)^l^	5036 ± 410 (106 ± 9)	5396 ± 549 (114 ± 11)^l^
	**2^nd^ Half**	5624 ± 543^b^^d^^e^ (114 ± 12)^b^^d^^e^	5078 ± 457 (102 ± 10)	5308 ± 376 (106 ± 8)	4627 ± 412 (94 ± 8)	5087 ± 471 (103 ± 10)	5362 ± 720 (107 ± 14)
	**Match**	11269 ± 689^b^^d^^e^ (116 ± 8)^be^	10149 ± 835 (104 ± 9)	10569 ± 863 (109 ± 9)	9369 ± 723 (97 ± 7)	10123 ± 764 (105 ± 8)	10758 ± 1142 (110 ± 11)

**High-Speed Distance (m)**	**1^st^ Half**	354 ± 102^b^ (7 ± 2)	248 ± 101 (5 ± 2)	255 ± 106 (5 ± 2)	270 ± 68 (6 ± 1)	337 ± 124 (7 ± 3)	450 ± 135^b^^c^^d^^e^ (9 ± 3)
	**2^nd^ Half Match**	347 ± 113 (7 ± 2)	267 ± 118 (5 ± 2)	253 ± 91 (5 ± 2)	254 ± 87 (5 ± 2)	349 ± 127 (7 ± 3)	449 ± 148^b^^c^^d^^e^ (9 ± 3)
	**Match**	701 ± 171^b^ (7 ± 2)	515 ± 195 (5 ± 2)	508 ± 169 (5 ± 2)	524 ± 110 (5 ± 1)	687 ± 222 (7 ± 2)	899 ± 259^b^^c^^d^^e^ (9 ± 3)

**Average Acceleration (m · s^-2^)**	**1^st^ Half**	0.63 ± 0.06	0.61 ± 0.05	0.65 ± 0.05	0.57 ± 0.02	0.62 ± 0.05	0.64 ± 0.06
	**2^nd^ Half**	0.59 ± 0.07	0.57 ± 0.07	0.61 ± 0.04	0.53 ± 0.04	0.59 ± 0.06	0.60 ± 0.07
	**Match**	0.61 ± 0.06	0.59 ± 0.05	0.63 ± 0.04	0.55 ± 0.03	0.60 ± 0.05	0.62 ± 0.06

Differences indicated if standardised mean difference is greater than 0.6. a = greater than AM, b = greater than CD, c = greater than DM, d = greater than STR, e = greater than WD, f = greater than WIN. 1 = greater than second half.

**TABLE 2:** Non-linear relationships of power law models. Data presented as mean ± 90% CI.

Variable			Attacking Midfielder	Central Defender	Defensive Midfielder	Striker	Wide Defender	Winger
**Relative Distance (m · min^-1^)**	**Intercept**	**1^st^ Half**	199 (188–211)	180 (159–205)	187 (163–214)	182 (157–211)	188 (165–215)	189 (166–216)
		**2nd Half**	190 (176–205)	178 (150–210)	185 (155–221)	177 (146–214)	186 (156–221)	188 (158–223)

	**Slope**	**1^st^ Half**	-0.17 (-0.18 – -0.16)	-0.17 (-0.19 – -0.15)	-0.16 (-0.18 – -0.14)	-0.18 (-0.20 – -0.17)	-0.18 (-0.20 – -0.17)	-0.18 (-0.19 – -0.16)
		**2^nd^ Half**	-0.16 (-0.19 – -0.14)	-0.18 (-0.23 – -0.13)	-0.17 (-0.22 – -0.12)	-0.20 (-0.25 – -0.14)	-0.19 (-0.24 – -0.14)	-0.19 (-0.24 – -0.14)

**Relative High-Speed Distance (m · min^-1^)**	**Intercept**	**1^st^ Half**	53 (44–63)	42 (28–63)	42 (27–65)	45 (28–72)	51 (33–78)	59 (39–90)
		**2^nd^ Half**	48 (37–62)	43 (24–76)	41 (23–75)	41 (21–79)	50 (28–91)	58 (32–104)

	**Slope**	**1^st^ Half**	-0.63 (-0.66 – -0.60)	-0.68 (-0.75 – -0.61)	-0.66 (-0.73 – -0.59)	-0.63 (-0.70 – -0.55)	-0.60 (-0.67 – -0.53)	-0.59 (-0.66 – -0.52)
		**2^nd^ Half**	-0.60 (-0.69 – -0.52)	-0.64 (-0.84 – -0.45)	-0.63 (-0.83 – -0.43)	-0.68 (-0.89 – -0.47)	-0.59 (-0.79 – -0.40)	-0.61 (-0.81 – -0.41)

**Average Acceleration (m · s^-2^)**	**Intercept**	**1^st^ Half**	1.05 (1.00–1.10)	1.02 (0.92–1.13)	1.06 (0.95–1.18)	1.01 (0.89–1.14)	1.07 (0.96–1.19)	1.05 (0.94–1.17)
		**2^nd^ Half**	1.01 (0.95–1.08)	0.97 (0.83–1.13)	1.02 (0.87–1.19)	0.94 (0.79–1.12)	1.01 (0.86–1.17)	1.03 (0.88–1.20)

	**Slope**	**1^st^ Half**	-0.18 (-0.18 – -0.17)	-0.17 (-0.19 – -0.15)	-0.17 (-0.19 – -0.15)	-0.19 (-0.21 – -0.17)	-0.18 (-0.20 – -0.16)	-0.17 (-0.19 – -0.15)
		**2^nd^ Half**	-0.18 (-0.20 – -0.15)	-0.17 (-0.23 – -0.12)	-0.19 (-0.24 – -0.13)	-0.19 (-0.25 – -0.13)	-0.18 (-0.24 – -0.13)	-0.19 (-0.25 – -0.13)

## DISCUSSION

The current study quantified the within- and between-halves and positional groups differences in peak match running demands for commonly assessed physical performance metrics. To provide further context, absolute measures of physical performance were also assessed. Both the absolute total and high-speed running demands were maintained between halves, however, the second half was on average ˜3 min longer, reducing the relative distance covered for CD, DM, STR and WIN (˜5–7 m · min^−1^). Conversely, the peak running demands of TD and HSD were similar between halves across all positional groups. Interestingly, while the total AveAcc was similar between halves for all positions, the peak AveAcc demands were reduced for DM, STR and WD in the second half. Taken together, the results demonstrate that the total and peak match demands of TD and HSD differ between positions but are maintained between halves. Separately, the peak AveAcc differ between positions and decline in the second half for some positions which provides direction for the prescription of training drills in conditioning for the high-energy demanding acceleration and deceleration actions.

While the quantification of match running volumes describes the global demands of match-play, it offers limited information to help guide conditioning and training drill prescription. The current peak match running demands ranged between 112–199 m · min^−1^ for relative TD and 8–59 m · min^−1^ for relative HSD, depending on position and epoch duration. These data are similar to those previously reported in the Australian A-League (TD: ˜115–205 m · min^−1^, HSD: ˜10–65 m · min^−1^) [13] and English Championship (TD: ˜115–197 m · min^−1^ HSD: ˜13–61 m · min^−1^) [14]. However, the AveAcc in the present study was considerably higher than that previously reported for the Australian A-League (0.60–1.07 m · s^−2^ vs 0.52–0.90 m · s^−2^) [13]. Additional studies have assessed AveAcc in youth football players, however, differences in data filtering processes mean the data are not able to be directly compared [31]. As such, establishing a standardised methodology for assessing the Ave-Acc demands of match-play is warranted in future research.

The data demonstrated that AM have the greatest peak match running demands for relative TD covered, with CD and STR having the lowest. This is similar to previous data from the Australian A-League that identified CD as having the lowest peak match running demands [13], with all midfielders have the greatest and attackers have the lowest peak match running demands [14, 31]. Conflicting findings have been also reported on the peak relative HSD running demands, with the current study reporting that WD, WIN and AM have the greatest peak match high-speed running demands, whereas past data had reported that STR and WIN had the greatest demands [13] or that there were no positional differences [14]. Lastly, there is limited comparable data on the peak AveAcc demands, with previous research reporting that positional groups were similar, except for WD which had the greatest peak match running demands of AveAcc [13]. The current data presented similar findings, with the exception for STR, which demonstrated the lowest AveAcc demands. It is likely that the lack of consensus amongst literature around which positional group has the greatest peak match running demands for HSD and AveAcc is due to the differing demands associated with different playing formations or team tactics [32, 33]. While this data in the present study may not be reflective of all teams and competitions, it provides rationale for evaluating peak match running demands of competition relative to positional group.

Positional discrepancies in match running demands may reflect several contextual factors related to the performance of high-intensity efforts, which impact on a player’s peak match running demands. High-intensity efforts are closely linked to critical parts of a match, such as scoring or defending goals [11, 12], and with more goal scoring opportunities in the second half [9, 10]**,** there are likely more instances where high-intensity efforts are performed to impact upon the match result. As such, while absolute running demands of relative TD are lower, absolute and peak match running demands of HSD are maintained. Furthermore, ball in play time is lower in the second half [34] due to the more frequent game interruptions, and as a result the time spent in lower locomotor speed categories increases [35]. Such a trend would result in a reduction in total physical output, but not impact on the peak match running demands. In comparison, any declines in peak AveAcc demands were position dependent, with AM, CD and WIN maintaining peak running intensities between halves, with DM, STR and WD declining. Past data has shown that the number of accelerations and decelerations performed in the second half reduces, with match related fatigue suggested as a contributing factor [36, 37]. While other factors such as self-imposed pacing strategies [3], or changes to team tactics [38] may also contribute. However, the collective data demonstrate that the peak AveAcc match demands are reduced in the second half for some positional groups.

While data is constrained by the limitation that it was only collected from a single professional football team, the assessment of positional peak match running demands is warranted to identify differences within a match, across all three assessed metrics. Hence, the application of such data should be to replicate these demands during training through the designing and implementation of position specific training drills. The methods presented by Delaney, Thornton [13] provide scope as to how to mathematically estimate peak match demands for a given duration using the data presented in Table 2. Exposure of players to peak match running demands in the initial phases of training will largely replicate the most difficult physical intensities of match-play, with peak demands typically greater in the first half. Conversely, structuring a training session to include match simulation drills at the end of a session may help develop the ability of players to perform at higher intensities during key tactical moments in the latter stages of a match. Further, while the underlying mechanistic properties behind differences in running performance between halves are multi-faceted, the understanding that reductions occur may help coaches evaluate implemented strategies aimed at mitigating said differences. Additionally, while not quantified in the present study, the technical and tactical demands of drills replicating match demands drills should also be considered, with drill dimensions, player numbers and drill constraints considerably impacting on both the technical and tactical demands, as well as physical demands [39]. Additionally, while peak match demands differed between halves for some positional groups, it is clear that players are regularly required to perform at or near peak match demands frequently across a match [19]. Hence, frequent performance at or near peak demands are suggested within training sessions. It is however important to note that peak physiological demands were unable to be quantified in the current study, with it possible that there may be a dissociation between peak physical and physiological demands, which would be useful for greater granularity when prescribing conditioning drills. Additionally, while not quantified in the current study, the variability of peak match running demands have previously been reported on, with the inherent variability of the measures worth considering [40].

Peak match running demands have emerged as a detailed method of assessing physical match performance that can aid in the design and prescription of training stimuli. As such, providing context surrounding the acute changes in peak match running demands is crucial in preparing players for competition. Overall, the between half changes to peak match running demands are position dependent, indicating that the assessment of acute changes in peak match running demands should be assessed on a positional basis, as opposed to a team basis. Further, with past data demonstrating that peak match intensities occur at various stages throughout a match [23], the timing of player exposure to these demands during training should be considered in developing training practices. Overall, the present study provides a framework in which to gauge the physical between half match performance of elite soccer players, in relation to peak match running demands.

## Disclosure Statement

No potential conflict of interest was reported by the authors.
